# Matrix Vesicles: Role in Bone Mineralization and Potential Use as Therapeutics

**DOI:** 10.3390/ph14040289

**Published:** 2021-03-24

**Authors:** Sana Ansari, Bregje W. M. de Wildt, Michelle A. M. Vis, Carolina E. de Korte, Keita Ito, Sandra Hofmann, Yuana Yuana

**Affiliations:** 1 Orthopaedic Biomechanics, Department of Biomedical Engineering, TU Eindhoven, 5600 MB Eindhoven, The Netherlands; s.ansari@tue.nl (S.A.); b.w.m.d.wildt@tue.nl (B.W.M.d.W.); m.a.m.vis@tue.nl (M.A.M.V.); c.e.d.korte@student.tue.nl (C.E.d.K.); K.Ito@tue.nl (K.I.); s.hofmann@tue.nl (S.H.); 2 Institute for Complex Molecular Systems, Eindhoven University of Technology, P.O. Box 513, 5600 MB Eindhoven, The Netherlands; 3 Department of Biomedical Engineering, TU Eindhoven, 5600 MB Eindhoven, The Netherlands

**Keywords:** bone mineralization, biomimetic, extracellular vesicles, matrix vesicles, osteoblasts, therapy

## Abstract

Bone is a complex organ maintained by three main cell types: osteoblasts, osteoclasts, and osteocytes. During bone formation, osteoblasts deposit a mineralized organic matrix. Evidence shows that bone cells release extracellular vesicles (EVs): nano-sized bilayer vesicles, which are involved in intercellular communication by delivering their cargoes through protein–ligand interactions or fusion to the plasma membrane of the recipient cell. Osteoblasts shed a subset of EVs known as matrix vesicles (MtVs), which contain phosphatases, calcium, and inorganic phosphate. These vesicles are believed to have a major role in matrix mineralization, and they feature bone-targeting and osteo-inductive properties. Understanding their contribution in bone formation and mineralization could help to target bone pathologies or bone regeneration using novel approaches such as stimulating MtV secretion in vivo, or the administration of in vitro or biomimetically produced MtVs. This review attempts to discuss the role of MtVs in biomineralization and their potential application for bone pathologies and bone regeneration.

## 1. Introduction

Bone is a multifunctional organ that is maintained through lifelong remodeling by the bone-forming osteoblasts, bone-resorbing osteoclasts, and the bone-regulating osteocytes. These cells reside on/in a composite matrix, comprising mainly the organic collagen type 1 and the mineral hydroxyapatite which are highly organized at multiple hierarchical levels [1]. This matrix and its organization give bones their remarkable mechanical properties and support their function as a storage for calcium and phosphate ions [2].

Osteoblasts are responsible for the creation of the mineralized organic matrix. They produce collagen type 1, which is considered to be the template for mineral nucleation and mineral crystal growth [3,4,5]. After mineral precursors have entered the collagen gap region, hydroxyapatite crystals grow outside the dimensions of the fibril, forming an interconnected continuous cross-fibrillar pattern [6]. This mineralization is believed to be regulated by osteoblasts via indirect and direct mechanisms [7]. The indirect mechanism involves the synthesis of negatively charged non-collagenous proteins which are believed to be associated with the collagen gap region where they may direct mineral precursors into the collagen fibril [8,9]. The direct mechanism is thought to be associated with extracellular vesicles (EVs) produced by osteoblasts [7,10]. 

Osteoblast-derived EVs, similar to other EVs produced by different cell types, are phospholipid-enclosed nanoparticles containing a variety of lipids, proteins and nucleic acids. There is increasing evidence that osteoblast-derived EVs have a multitude of functions, including the promotion of osteogenic differentiation, inducing osteoclast formation, and mineralization of the organic matrix [7,10,11,12,13,14,15,16,17]. In general, vesicles involved in matrix mineralization are referred to as matrix vesicles (MtVs); they can bind to the collagen matrix and are equipped with mineralization-specific components such as phosphatases, calcium, and inorganic phosphate [10,18,19]. These calcium and phosphate ions can remain amorphous or can crystalize inside the MtVs into hydroxyapatite. Upon crystallization, they can break through the vesicle’s membrane to form a mineral nodule [20]. 

Besides MtVs’ essential role in mineralization of the organic bone matrix, they also have shown innate osteo-inductive properties [17]. In addition, chondrocyte- and osteoblast-derived MtVs are involved in endochondral ossification; a process which is part of natural bone regeneration [21]. This indicates their therapeutic potential for bone pathologies and fracture healing. Accordingly, osteoblast-derived EVs, from which MtVs are likely a significant part, were already proposed as a promising therapeutic for osteoporosis and fracture healing [17,22,23,24]. 

In this review article, the roles of EVs—and more specifically, MtVs—in bone and the mineralization of the organic matrix are summarized. Current methods for the isolation and characterization of MtVs are discussed. An overview of the current therapeutic applications of MtVs for bone disorders, and possible future applications of (biomimetic) MtVs, are presented.

## 2. EV Subtypes, Biogenesis, and Their Biological Potentials

The term “extracellular vesicles” was chosen in September 2011 as a generic term by the International Society for Extracellular Vesicles (ISEV), a group of scientists with collective long-term expertise in the field of EV biology [25]. EVs stand for particles naturally released or secreted from prokaryotic and eukaryotic cells that are delimited by a lipid bilayer [25,26,27]. The first use of an “extracellular vesicle” in the title of a scientific publication was in 1971, when Aaronson et al. showed that the eukaryotic alga *Ochromonas danica* could produce a large variety of small and large intra- and extracellular membrane-bound vesicles. These vesicles were recovered in the centrifugates of the cell-free *Ochromonas danica* after ultracentrifugation [28]. In 1967, Peter Wolf had found that fresh plasma freed of intact platelets through ultracentrifugation contained particulate material which he called platelet-dust. This platelet-dust was the first scientific appearance of platelet-derived EVs [29].

EVs are found in most biological fluids; for example, cell culture supernatant, blood, urine, saliva, amniotic fluid, milk, synovial, and seminal fluids [30,31,32]. Furthermore, EVs can be extracted from tissues such as brain, tumor, bone, and cartilage [21,33,34,35,36]. In general, EVs are heterogenous in their size and morphology, and they can contain a variety of organic and inorganic cargoes such as lipids, proteins, genetic materials (DNA/RNA), and minerals derived from their parent cells [20,25,26,37]. 

### 2.1. Biogenesis of EVs

**Figure 1:**
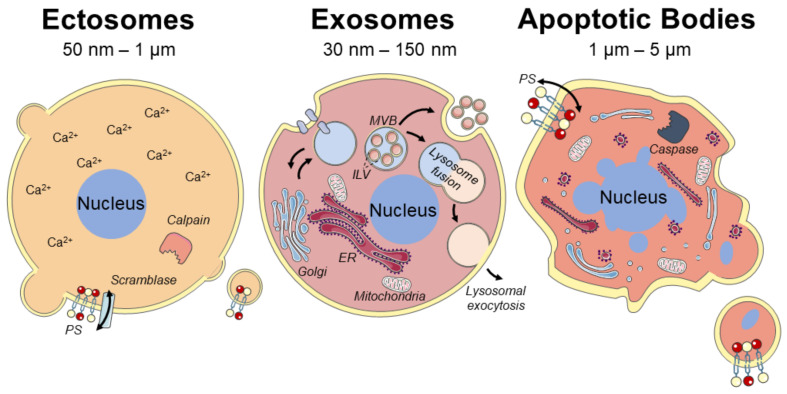
Subtypes of extracellular vesicles (EVs) based on their possible biogenesis pathways. EVs can appear as ectosomes that bleb from the cell membrane, as exosomes that are formed inside the cell after endocytosis, or as apoptotic bodies that derive from cells undergoing apoptosis. MVB, multi vesicular body; ILV, intra luminal vesicle; ER, endoplasmic reticulum; PS, phosphatidylserine. The figure was modified from Servier Medical Art, licensed under a Creative Common Attribution 3.0 Generic License (http://smart.servier.com/, accessed on 20 January 2021).

Consensus has not yet emerged on specific markers of EV subtypes; therefore, assigning an EV to a particular biogenesis pathway remains a challenge. Nevertheless, based on the current knowledge in the field, EVs can be classified into three subtypes: (i) plasma membrane-derived ectosomes (shedding microparticles/microvesicles); (ii) endosome-originated exosomes; and (iii) apoptotic bodies (Figure 1) [32,37].

#### 2.1.1. Plasma Membrane-Derived Ectosomes

Ectosomes are produced via the outward budding of plasma membranes [32,38]. These vesicles are often generically referred to as microvesicles/microparticles, although they are extremely heterogeneous in size, ranging from exosome-like EVs of 50 nm to microvesicles as large as 1 μm [37,38,39]. An increased influx of calcium ions in the cell, which may be triggered after a plasma membrane injury, seems to initiate the biogenesis of ectosomes and generate heterogeneity in their sizes and cargoes [40,41]. The increase in cytosolic calcium concentration can activate the proteins scramblase and calpain [42]. The activation of scramblase leads to a loss of membrane phospholipid asymmetry and exposure of the negatively charged phospholipid phosphatidylserine (PS) [43]. The activation of calpain leads to the calcium-dependent degradation of various proteins, allowing the outward budding of ectosomes from the plasma membrane [38,43,44]. Additionally, an increase in cytosolic calcium due to plasma membrane injury may trigger the endosomal sorting complex required for transport (ESCRT) machinery, which in turn results in ectosomes being released during excision of the injured membrane [40,45,46,47].

#### 2.1.2. Endosome-Originated Exosomes

The biogenesis of exosomes begins with the inward budding of small parts of the plasma membrane containing several membrane protein components and the formation of early endosomes. Upon inward budding of the plasma membrane, the extracellular proteins/molecules are encapsulated into small vesicles. These vesicles fuse with early endosomes which serve as the focal point of the endocytic pathway. At this stage, there is also continual fusion of transport vesicles containing newly synthesized molecules from the trans-Golgi network. The early endosomes then become mature and transform into late endosomes [48,49]. Molecules are also sorted into smaller vesicles that bud from the perimeter membrane into the endosome lumen, forming intraluminal vesicles (ILVs). This leads to the multivesicular appearance of late endosomes known as multivesicular endosomes or multivesicular bodies (MVBs) [50,51]. When molecules are destined for degradation inside the cell, MVBs fuse with lysosomal membranes and release ILVs into lysosomes for degradation. In some cases, for instance when there is an increased influx of calcium ions into the cytosol due to plasmalemmal damage and tissue remodeling, lysosomes can also move towards the plasma membrane, fuse, and release their intraluminal vesicle cargoes [52]. When molecules are destined for recycling outside the cell, instead of fusing with lysosomes, MVBs can also fuse with the associated plasma membrane. ILVs are then released extracellularly as exosomes, small EVs with a diameter of around 30–150 nm, which carry the cargoes of the MVBs [31,53]. What defines the choice for degradation or recycling of the cargoes of MVBs is unknown, and is likely related with the multiple machineries proposed in the biogenesis of ILVs for MVBs [32,54]. The ESCRT is the most extensively described pathway of MVB biogenesis, responsible for the sorting of ubiquitinated proteins into ILVs [51,53]. Several ESCRT-associated proteins such as PDCD6IP, tumor susceptibility gene 101, and heat shock protein 70 have been used to identify exosomes [31,53,55,56,57,58,59,60]. Interestingly, sorting of exosomal cargoes into MVBs can also occur in an ESCRT-independent manner, which seems to be driven by the presence of certain lipids, such as lysobisphosphatidic acid and ceramides, within the endosomal membrane [53,61]. These lipids might organize into specialized endosomal regions, bend inward, and ultimately form vesicles enriched in tetraspanins such as CD9, CD63, CD81, and CD82 [61]. Whether each pathway acts in different MVBs, or if they simultaneously act on the same MVB, is not known.

#### 2.1.3. Apoptotic Bodies (ApoBDs)

Unlike other types of EVs such as exosomes and ectosomes, ApoBDs with sizes between 1 and 5 μm are generated only via budding of the plasma membrane of cells undergoing apoptosis which include caspase activation and DNA fragmentation [62,63,64]. Like ectosomes, ApoBDs expose PS on the outer leaflet of their membrane and retain cell type-specific markers [62,64]. It is thought that ApoBDs are released during the late stages of cell death, whereas ectosomes are released during the early stages of apoptosis [42,62,65]. Some ApoBDs, thus, contain DNA and nuclear proteins such as histones, which can be useful markers to distinguish ApoBDs from other EV subtypes [62,64].

### 2.2. Biological Potentials of EVs

Although initially considered as inert cellular debris, EVs are now recognized as being important mediators in intercellular communication and many biological processes [31,32,66]. It has been shown that the concentration, composition, and cellular origin of EVs in body fluids differ between healthy subjects and patients suffering from diseases such as cancer, and cardiovascular and inflammatory diseases [31,67,68,69,70,71,72]. Therefore, there is a growing scientific and medical interest in EVs as valuable biomarkers in diagnostics and as therapeutics. 

#### 2.2.1. Use of EVs in Diagnosis

EVs are present in various body fluids at relatively high numbers—for example, blood plasma contains more than 10,000 EVs per milliliter—therefore, their enumeration in body fluids offers quantitative advantages [73]. Furthermore, obtaining body fluids to isolate EVs for diagnostic purposes is less invasive and relatively low-cost compared to obtaining tissue biopsy. Thus, many clinicians and biotechnology companies are attracted to research and develop EVs as a “liquid” biopsy [74]. One of commercialized EV-based diagnostics is ExosomeDx™. This is a non-invasive urine test for prostate cancer initially developed by Exosome Diagnostic, and now commercialized by Bio-Techne [75].

#### 2.2.2. Use of EVs for Therapy

EVs are naturally derived from cells and carry various molecules and lipids which are targeted at other specific cell types. This indicates that EVs are exploitable for therapeutic purposes to target specific cells or tissue components [76,77,78,79]. Similar to nanoparticles, EVs (particularly exosomes and ectosomes) are in the nanometer range; they can be loaded with drugs or other inorganic particles [79,80,81,82,83]. The added value of EVs as drug nanocarriers is that EVs are not foreign to the host immune system because they are naturally derived. Therefore, EVs would overcome the issues of toxicity as well, which can occur when synthetic biomaterial nanoparticles are used to deliver drugs to target cells [84]. Recently, the use of EVs as therapeutic nanocarriers in the clinical settings have been reviewed [85,86,87,88,89,90]. One example of a successful EV-based therapeutic is Bexsero^®^, a meningitis serogroup B vaccine, which was approved in 2013 by the European Medicines Agency and the U.S. Food and Drug Agency. This vaccine contains EVs, referred to as outer membrane vesicles (OMVs), derived from Gram-negative bacteria *Neisseria meningitidis*. This vaccine is used for the prevention of meningococcal disease caused by *Neisseria meningitidis* group B bacteria in individuals from two months old through to 25 years of age [79,91].

### 2.3. EVs Derived from Bone Cells

The skeletal system functions and maintains itself based on communication between cells of various origins. Osteoblasts and osteoclasts, responsible for bone formation and resorption, respectively, are of importance for bone homeostasis. Thus far, the communication between osteoblasts and osteoclasts has been suggested to occur at the protein level via a direct contact (i.e., membrane-bound ligands and gap junctions), secreted cytokines, and deposited growth factors in the bone matrix [12,92]. However, it has also been reported that osteoblasts and osteoclasts can communicate via an indirect contact through EVs. Such communication regulating bone remodeling can, for example, happen through the interactions with ligands that are present on the EV’s surface or by transferring EV cargoes exchanging EV enclosed genetic information, such as microRNAs (miRNAs) [22,92,93,94,95,96].

Cappariello et al. found that osteoblasts pre-treated with parathyroid hormone (PTH) generated EVs carrying the receptor activator of nuclear factor kappa-B ligand (RANKL) and demonstrated that these EVs supported the survival of osteoclasts in vitro [22]. In vivo, intraperitoneal injection of EVs from wild-type osteoblasts into RANKL−/− mice lacking tartrate-resistant acid phosphatase (TRAP) expression increased the presence of TRAP-positive cells in trabecular bone, which is indicative of neo-osteoclastogenesis [22,97]. Mature osteoblasts also release EVs with specific characteristics involved in matrix mineralization. These EVs are anchored to protein components of the surrounding extracellular matrix and are known as MtVs [20,98]. Their contribution to bone mineralization is discussed in Section 3.

Osteoclasts and their precursors have also been described to generate EVs with a diameter between 25 and 120 nm, similar to the size of exosomes [96]. Their membranes are enriched with epithelial cell adhesion molecule, CD63, and RANK. Interestingly, RANK-rich osteoclast-derived EVs act as inhibitors of osteoclastogenesis through competitively decreasing the RANK–RANKL interaction with, e.g., osteoblasts [96,99]. During bone remodeling, osteoclasts undergo apoptosis at the end of the bone resorption phase, and produce large amounts of ApoBDs [100]. These vesicles promote osteogenesis via RANKL reverse signaling. These studies show that the maintenance of bone homeostasis relies heavily on cellular communication between osteoclasts and osteoblasts through the RANKL interactions; EVs are likely to play a role in this process. 

It also has been shown that communication between osteoclasts and osteoblasts is mediated by the transfer of miRNAs which are contained within EVs. Through direct incubation of osteoclast-derived exosomes containing miR-214 with osteoblasts in vitro, osteoblast activity was inhibited [95,101]. In the case of osteoblasts, 43 miRNAs were found to be highly abundant in mineralized exosomes of the MC3T3-E1 pre-osteoblast cell line [93]. Those osteogenic miRNAs were able to promote bone marrow stromal cell (ST2) differentiation into osteoblasts [93]. Osteoblasts released EVs carrying miR-125b and miR-503 with anti-osteoclastogenic activity [102,103]. Interestingly, it has been shown that EVs carrying miR-125b could prevent bone loss in a mouse model of post-menopausal osteoporosis [102]. Thus, EVs carrying miR-125b could be able to inhibit bone resorption, emphasizing the role of EVs in bone remodeling. 

Osteocytes, the most abundant cellular component of mature bone, are terminally differentiated osteoblasts, which reside deep within the bone matrix [104]. The osteocytes orchestrate the actions of both osteoblasts and osteoclasts through relaying external mechanical signals, to trigger the deposition or resorption of bone possibly via the expression osteoprotegerin (OPG) and RANKL [105]. It has been shown that osteocyte mechanosensitivity is encoded through unique intracellular calcium dynamics [41]. Upon fluid flow, osteocytes showed a transient increase in intracellular calcium ions and these cells released a substantial amount of EVs containing bone regulatory proteins such as sclerostin, RANKL, and OPG into the culture medium [41]. Osteocytes also released EVs containing miR-218, which inhibited sclerostin and influenced the differentiation of osteoblasts [106]. Interestingly, the miR-218 contained in these EVs can be suppressed by myostatin secreted by muscles [106]. These results indicate possible bone–muscle communications.

Summary of the bone cell populations producing EVs including their cargoes and functions can be found in Table 1. 

## 3. MtVs and Their Contribution to Bone Mineralization

MtVs are a subgroup of EVs. They were first detected in cartilage and bone through electron microscopy methods [33,34,107]. They range in size from 10–400 nm and are considered to derive from mineralization-mediating cells such as chondrocytes, osteoblasts, and odontoblasts [21,35]. Similar to EVs, their biogenesis can most likely be through multiple distinct pathways. Therefore, unlike other types of EVs (e.g., exosomes or ectosomes), MtVs cannot simply be classified based on their size or biogenesis mechanism. Instead, MtVs are normally referred to by their function and location in the extracellular matrix of mineralized tissues.

Most MtVs are electron-dense and equipped with mineralization supportive proteins [10,18,108,109,110]. It is believed that these MtVs accumulate calcium and inorganic phosphate and, upon supersaturation, precipitate into needle-like hydroxyapatite [Ca^10^(PO^4^)^6^(OH)] crystals whose growth eventually disrupts the MtVs’ membrane [35,109]. Under transmission electron microscopy (TEM), these crystals feature spicules or a ribbon-like structural profile, approximately 25 nm wide, 10 nm high, and 50 nm long [20]. Subsequently, their growth can continue in the extravesicular space to form more stable crystals, or to propagate on the collagen fibrils [18,35,111]. Some MtVs are electron-lucent and are referred to as empty MtVs [112,113]. Whether these MtVs will still gradually accumulate calcium and phosphate over time or whether they remain electron-lucent is unknown.

### 3.1. MtV Biogenesis and Characteristics

**Figure 2:**
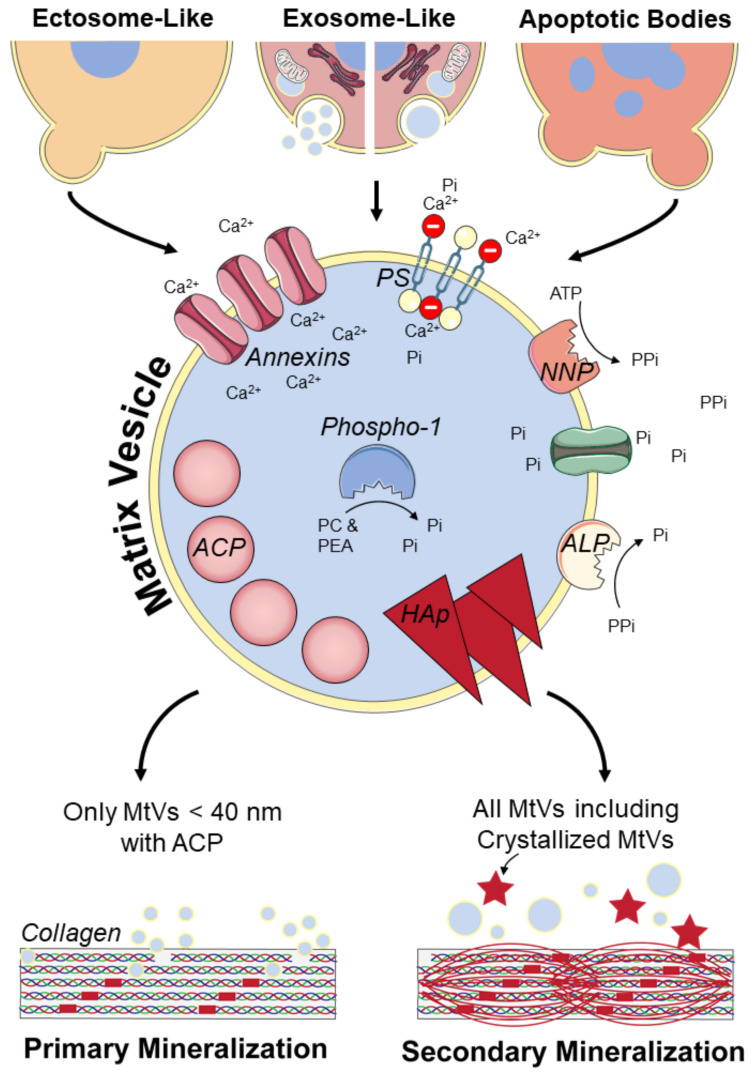
Possible MtV biogenesis pathways and collagen mineralization mechanisms. MtV, matrix vesicle; ALP, alkaline phosphatase; NNP, nucleotide pyrophosphatase; PS, phosphatidylserine; ACP, amorphous calcium phosphate; HAp, hydroxyapatite. The figure was modified from Servier Medical Art, licensed under a Creative Common Attribution 3.0 Generic License. (http://smart.servier.com/, accessed on 20 January 2021).

Currently, MtVs are often only described as microvesicles or ectosomes which are formed by budding from the plasma membrane of their parent cell [21,35]. However, once MtVs are released from their parent cell, assigning them to a distinct biogenesis pathway is extraordinarily difficult unless the vesicle is caught in the act of being released; for example, by live imaging techniques [25]. Multiple MtV biogenesis mechanisms have been reported. Probably, these different biogenesis mechanisms also will result in some specific characteristics of the MtVs. However, most studies only focus on the biogenesis or the characterization of MtVs, making it difficult to couple specific MtV characteristics to a specific biogenesis pathway. Here, we describe the characteristics, as reported in the literature, for the different biogenesis pathways of MtVs (Figure 2 and Table 2).

#### 3.1.1. Ectosome-Like MtVs

There is morphological evidence indicating that some MtVs are formed by budding and then pinching off from the plasma membrane of cells mediating mineralization [21,35]. To be able to accumulate calcium and inorganic phosphate, the membranes of these MtVs are enriched with mineralization-related proteins (Figure 2). These include PS-binding annexin proteins and phosphatases [18,114,115,116,117]. Alkaline phosphatase (ALP) on the MtV’s membrane, which is also present on the membrane of osteoblasts, dephosphorylates pyrophosphate [18]. Phosphate transporters can subsequently transfer free phosphate into the MtVs [18]. Nucleotide pyrophosphatase-1 (NNP-1) can also be found on the plasma membrane of MtVs and their parent cells. This enzyme frees pyrophosphate from adenosine triphosphate (ATP), indirectly providing a new phosphate source for the MtV [118]. However, pyrophosphate also functions as an inhibitor of mineralization [119]. Therefore, ALP and NNP-1 are believed to control the pyrophosphate/inorganic phosphate ratio that is needed for physiological matrix mineralization [18]. In addition to these phosphatases on the MtV’s membrane, Phospho1 inside the vesicle might be involved in MtV mineralization by producing phosphate from phosphocholine and phosphoethanolamine [118,120]. Besides these phosphatases, the high level of PS on the plasma membrane of MtVs indicates that PS is an important contributor to the mineralization process as well; their potential function is described in Section 3.2. [42]. Annexin proteins (annexin A2, A5, and A6) are also present at substantial concentrations on the MtV membrane [114,121,122]. Annexin A5, which can strongly bind to PS, increases the permeability of MtVs for calcium [114,121]. However, the function of annexins seems to not be critical for mineralization; bone development was not impaired in annexin A5 and A6 knockout mice [123,124]. 

#### 3.1.2. Exosome-Like MtVs

More recently, evidence was found for the formation of MtVs intracellularly through an exosome-like mechanism [125,126,127]. This process was first described by Boonrungsiman et al., who found that there is transport of calcium and phosphate ions from mitochondria to intracellular vesicles that support mineralization [7]. These mitochondria are likely provided with calcium and phosphate from the endoplasmic reticulum [127]. The amorphous calcium phosphate-containing mitochondria can fuse with lysosomes, becoming autolysosomes, where they subsequently undergo mitophagy [125,127]. Within the lysosomal compartment, membrane components of the mitochondria are degraded, and amorphous calcium phosphate is freed in the autolysosome which could be released via exocytosis in the extracellular matrix as an MtV [125]. Due to the acidic environment within lysosomes, amorphous calcium phosphate crystallization is prevented [126]. Thus, lysosomes seem to fulfil the role of intracellular transporters of amorphous calcium phosphate-containing MtVs to the plasma membrane [126]. At early stages of osteoblastic differentiation, vacuolization of mitochondria was observed, probably leading to the formation of MVBs, and thus smaller and multiple MtVs from one mitochondrion [126,127]. Whether these exosome-like MtVs could be identified upon release from their parent cell and how exactly they differ from the ectosome-like MtVs described earlier needs more detailed investigation.

#### 3.1.3. ApoBDs Involved in Mineralization

Mineralization often also occurs with apoptosis. For example, in growth plates where terminally differentiated chondrocytes undergo apoptosis, this process results in the formation of ApoBDs [121]. This is also thought to be an important initiator of vascular calcification [128]. These ApoBDs are also able to accumulate calcium and phosphate, but they contain only a few calcium channel-forming annexins [121]. Unlike ectosome-like MtVs, ApoBDs do not need annexins to induce mineralization, because they do not accumulate calcium inside the vesicle [129]. ApoBDs are rich of externalized PS, which could function as mineral nucleation sites because they can stabilize calcium and phosphate [130]. Thus, ApoBDs seem to accumulate calcium and phosphate on their membrane. 

ApoBDs also seem to influence mineralization indirectly, by enhancing ALP and inhibiting NNP-1 exposure on the membranes of endplate chondrocytes [131]. This results in more available inorganic phosphate, which promotes mineralization [131]. Taken together, because MtVs exhibit multiple characteristics that are involved in calcium and phosphate accumulation, all MtVs may contribute to matrix mineralization regardless of their biogenesis pathway. Our findings on the different types of MtVs and their physical, biological, and functional properties as described in the previous sections are summarized in Table 2.

### 3.2. Potential Collagen Mineralization Mechanisms

MtVs can bind to the collagen matrix through, e.g., annexins and ALP [19]. How MtVs subsequently interact with the collagen matrix and how this leads to intra- or extrafibrillar mineralization, is still largely unknown. Most likely, primary intrafibrillar mineralization of newly formed collagen is regulated by MtVs smaller than 40 nm containing amorphous calcium phosphate, which could enter the collagen gap region (~40 nm) (Figure 2) [7]. These vesicles were found to be secreted by mid-to-late stage differentiated osteoblasts [17]. In the later stage of osteogenic differentiation, needle-like crystals were found, probably matured MtVs from which the membrane was disrupted by the crystals [17]. These membrane-free crystals were less able to induce mineralization than the vesicles with amorphous calcium phosphate [17]. This indicates that the vesicle’s membrane has an important role in inducing primary or intrafibrillar mineralization (i.e., in the collagen gap region), and that these crystallized MtVs probably only support secondary or extrafibrillar collagen mineralization (Figure 2). In this regard, ectosome-like MtVs and mineralizing ApoBDs are rich with externalized PS, creating a more negatively charged vesicle membrane [16,42]. This PS on the vesicle’s membrane can stabilize calcium and phosphate [130]. Interestingly, negatively charged non-collagenous proteins are believed to form these complexes as well, and their importance for mineralization is indisputable [9]. This might suggest that externalized PS could help, as non-collagenous proteins do, to guide MtVs to the collagen gap-region [9]. Most likely, MtVs larger than 40 nm also contribute to secondary collagen mineralization, although their exact role has yet to be elucidated. Thus, MtVs can vary in (membrane) composition, calcium phosphate crystallinity, and size, depending on the biogenesis of the vesicle and the differentiation stage of their parent cell. Probably, their function to induce primary intrafibrillar or secondary extrafibrillar mineralization is defined by these characteristics.

## 4. Isolation and Characterization of MtVs

To understand the role of MtVs during the mineralization process, attempts have been made to isolate these vesicles, separate them from other EVs, and characterize them based on their physical and biological properties and their functionality. The approaches to isolate and characterize MtVs are discussed in this section. 

### 4.1. Isolation of MtVs

For the isolation of MtVs, centrifugation-based methods are commonly used. Around two-thirds of the reviewed papers which included MtV isolation in their methodology used differential centrifugation (DC) combined with a final ultracentrifugation (UC) (>10,000× *g*) step [17,102,107,132,133,134]. Even though DC is most often used to isolate MtVs, the exact execution differs in the number of steps, centrifugation speed, and centrifugation time used. Other common strategies for isolating MtVs include DC/UC in combination with ultrafiltration [23,135,136,137,138,139]. Size-exclusion chromatography, fluid flow fractionation, polymer precipitation, immunoaffinity isolation, and microfluidic-based technologies are often used to isolate and/or concentrate EVs, although none of these techniques has been reported to be used for the isolation of MtVs [140]. The size and some biological characteristics of MtVs overlap with those of other EVs; therefore, the isolation techniques used for EVs may also be suited for isolating MtVs.

Hutcheson et al. hypothesized that time-dependent UC may enrich mineral-containing vesicles more than other vesicular populations, because of their greater physical density [141]. They found electron-dense vesicular structures to be associated with hydroxyapatite crystals with high ALP activity and mineralization potential in the pellet after only 10 min of UC of the conditioned media of calcifying coronary artery smooth muscle cells. Significantly, greater amounts of protein were pelleted after 10 min UC for the calcifying samples compared to the control samples. If the amount of protein per vesicle in both samples is approximately constant, they estimated that vesicles in the calcifying samples were 35% more dense than those in control samples. Shortening the time of UC decreased the contamination of other vesicle populations, but the number of calcifying vesicles was not increased. Hutcheson et al. suggested performing collagenase digestion of the cell culture prior to UC to increase the yield of MtVs [141].

Collagenase breaks down collagen fibers and is believed to release the MtVs that are embedded in the matrix [142,143]. Therefore, to isolate MtVs from tissue, a digestion step with collagenase should be performed prior to MtV isolation [107,132,133]. MtVs can also be isolated from cell culture samples [17,102,144]. Some of the literature reported that a collagenase digestion step was added to release MtVs or mineralizing-EVs from the cell culture matrix [102,134,145,146,147]. To distinguish MtVs isolated from the cell culture matrix with the use of collagenase from MtVs isolated from the cell culture supernatant, the vesicles are sometimes referred to as collagenase-released matrix vesicles and medium matrix vesicles, respectively [35,148]. The effect of collagenase digestion on EV characteristics still needs thorough investigation. There are indications that collagenase treatment prior to EV isolation results in an isolated EV population with higher mineralization potential, as indicated by more apatite-like mineral, higher ALP activity, and higher cholesterol/lipid content than isolation without collagenase treatment [148,149]. Thus, collagenase treatment does not only improve the harvest of total EVs, but also yields MtVs with better mineralization potential. 

### 4.2. Characterization of MtVs

In 2018, the ISEV introduced guidelines for EV experimental research [25,26]. They recommend that for the preparation of EVs: (i) quantitative measures of the source are given; (ii) the number of EVs is estimated; (iii) the sample is tested for the presence of specific markers associated with EVs and EV subtypes; and (iv) the sample is tested for the presence of non-vesicular, co-isolated components. In this section, we discuss the characterization of MtVs based on these recommendations. Methods of characterization of MtVs are based on physical properties, biological properties, and functionality. There is no single perfect characterization method of MtVs; therefore, a combination of different methods is necessary. 

#### 4.2.1. Physical Properties

To increase the reproducibility of EV experiments and to allow for comparisons between studies, the source of the EVs should always be described in detail. For tissue-derived MtVs, the tissue type, its origin, condition, and preferably also the size and volume, are reported. When MtVs are collected from cell culture, a more elaborate source description is required. This includes the cell source, the number of secreting cells, cell passage, the types of culture medium, and the time between the last medium refreshing and EV isolation. MtVs can be obtained both from the culture supernatant or from the matrix with the use of collagenase; therefore, the isolation technique used needs to be described in detail in the methods section.

Besides the source of MtVs, their physical properties such as number, size, and morphology need to be reported. Nanoparticle tracking analysis (NTA) [41,134,136,137,143,150], dynamic light scattering (DLS) [23,135], and tunable resistive pulse sensing (TRPS) [139,151] can be used to determine particle size and bulk concentration. MtVs, like other subtypes of EVs, are heterogenous in size, and some non-vesicular contaminants such as protein aggregates may be co-isolated and interfere with measurement; therefore, these techniques are not optimal for characterizing a single MtV and measuring the concentration of MtVs.

Singular vesicle size, topology, and morphology have been studied using atomic force microscopy (AFM) [135]. AFM has also been used in combination with antibody-coated surfaces to capture specific subsets of EVs and simultaneously characterize the single vesicle size and topology/morphology. This approach has been shown for the characterization of specific subsets of EVs derived from platelets [39]. It also has been demonstrated that AFM operated in peak force quantitative nanomechanical property mapping (AFM-PFQNM) was able to measure the nanomechanical and morphological properties of individual MtVs under both mineralizing (with addition of calcium ions) and non-mineralizing fluid conditions [152]. TEM is a common method to characterize the physical properties of MtVs, including the size and the crystals present inside MtVs [17,23,134,144,149]. When the characterization of certain surface proteins on MtVs is needed, immunogold labeling of MtVs prior to TEM can also be performed. This method was used, for example, by New et al., to visualize the release of CD68-positive MtVs from macrophages [153]. 

#### 4.2.2. Biological Properties

The concentration of the total MtV can be estimated by measuring the total protein content of the sample. This is often assessed by a bicinchoninic acid (BCA) protein assay [93,128,135]. Similar to other subtypes of EVs, MtVs carry transmembrane proteins such as CD9, CD63, and CD81 [25,26,136,139]. The presence of these proteins can be demonstrated with Western blotting and flow cytometry [23,132,148]. Besides general membrane proteins, other markers are used to characterize MtVs. The MtV membrane contains a high concentration of PS, which can be detected with flow cytometry by binding to annexin A5 conjugated to a fluorescent probe [138]. A high concentration of PS does, however, not mean that the isolated vesicles are indeed MtVs, because ectosomes and ApoBDs are generally rich in PS [154]. MtVs can also be characterized using markers which are present on their source cells. Nahar et al. found that MtVs from chondrocytes contain several annexins (A1, A2, A4, A5, A6, A7 and A11), BMPs (1–7), VEGF, osteopontin, osteocalcin, osteonectin, and bone sialoprotein [132]. Even though most markers will not be MtV-specific, combining several markers helps to better characterize MtVs. ISEV also recommends checking isolated EV samples for the presence of non-vesicular components which could be co-isolated [25]. There have been several markers used to exclude non-vesicular components in MtV isolates such as GM130, which can normally be found at the Golgi membrane, β-tubulin as a cytosolic marker, and histone 1 as a nuclear marker [17,155].

#### 4.2.3. Functional Properties

MtVs can be identified based on their distinct functionality such as their ALP activity, and calcium and phosphate levels. The ALP activity from a sample of isolated vesicles can be measured with a *p*-nitrophenyl phosphate substrate [148]. The calcium and phosphate levels within the vesicles can also be determined. Calcium levels within MtVs can be measured by a colorimetric analysis, whereas phosphate levels can be measured by a modified version of the method defined by Ames using ascorbic acid [121,129,156,157]. 

A unique property of MtVs is their ability to form crystals and to mineralize collagen. An in vitro mineralization assay to study the calcification potential of MtVs can be performed by adding MtVs to a collagen type 1 coated culture dish containing a calcification medium comprising 15% fetal bovine serum (FBS) and 10 mM β-glycerophosphate [158,159]. However, it should be considered that FBS may contain ALP as well that might interfere with the mineralization assay results. Y. Kunitomi et al. embedded MtVs in a collagen hydrogel and incubated this gel in medium containing only 10 mM β-glycerophosphate [144]. The MtVs mineralized the collagen in the gel, but mineralization was more successful after fragmentation of the MtVs [144]. After a set incubation time, the amount of deposited calcium could be measured colorimetrically by the *O*-cresolpthalein complexone method [147]. Another slightly different method was used by Chaudhary et al., where isolated MtVs were incubated with calcium chloride and ATP as a phosphodiester substrate in a calcifying solution for 5.5 h [134]. The assay demonstrated that MtVs accumulate calcium ions from the extravesicular environment in a dose-dependent manner. Thus, these functional assays are useful.

## 5. Therapeutic Potential of Osteoblast-Derived EVs and MtVs

EVs are considered complex biological mediators of tissue development and regeneration that may feature innate therapeutic potential for diseases/regeneration [10]. At present, research into the therapeutic application of EVs, and particularly MtVs, to treat bone diseases is in its infancy. This might be due to the complexity of MtV components and technical difficulties in the isolation and characterization of MtVs [17]. Only few bone-related clinical trials are registered, as revealed by a search in the United States National Library of Medicine [160]. Two clinical studies on EVs are documented, one on bone inflammation and one on osteoarthritis [161,162]. Unfortunately, clinical trials on MtVs were not found. Nevertheless, interest is rapidly growing, with several publications outlining the potential therapeutic utility of EVs for the regeneration of a wide range of tissues, including bone [10].

Strikingly, therapeutic bone-related studies generally mention EVs, but not particularly MtVs. In fact, EVs comprise MtVs, and therefore these studies presumably included MtVs without characterizing them. Wei et al. specifically investigated the proportion of MtVs in EVs released by mineralizing MC3T3-E1 osteoblast precursors at different stages of differentiation using TEM. Their study showed that MtVs accounted for a considerable proportion of EVs, and MtVs derived at different stages showed varying sizes and crystallinities [17]. 

### 5.1. Cellular Source of MtVs for Therapeutics

Various cell types can secrete MtVs with the potential to induce mineralization. Osteoblasts, chondrocytes, and odontoblasts are involved in biomineralization, and vascular smooth muscle cells (VSMCs) and macrophages could stimulate pathological calcification [163,164]. MtVs secreted by these cell types show similar characteristics, such as the PS membrane containing annexin A2, annexin A5, and phosphatases, and the formation of hydroxyapatite inside the vesicles [146,153,165]. However, many other components/cargoes in MtVs likely affect their targeting potential. 

Many studies have used mesenchymal stromal cell (MSC)-derived EVs [139,166]. However, the advantages and disadvantages of a cellular source of EVs for certain therapeutic applications has not been investigated thoroughly [17,167,168]. It is probable that the cellular source of EV is essential for targeting certain tissue. For example, EVs derived from mineralizing osteoblasts were shown to possess innate bone-targeting potential [17]. These EVs were successfully labeled with fluorescent PKH67; when injected into the tail vein of mice, the fluorescence intensities of EVs in the femurs were strongly visible after two hours [17]. Thus, the use of osteoblast-specific EVs might be a more effective choice, because it has shown to better target bone tissue.

### 5.2. Potential Targets of MtVs for Bone Mineralization Disorders

Most studies that report osteoblast-derived EVs focus on osteoporosis or fracture healing [22,23,24]. For example, intravenous injection of EVs derived from osteoblasts in an osteoporotic mouse model significantly lowered bone loss as measured with micro-computed tomography [17]. In addition, the same study showed the osteo-inductive potential of MtVs visible by the induced expression of the osteogenic-related genes runt-related transcription factor 2 (RUNX-2), collagen type 1, and ALP in mesenchymal stromal cells cultured under growth conditions [17]. This osteo-inductivity makes MtVs an interesting target for bone regeneration applications.

Next to fracture healing and osteoporosis, mineralization of bone’s organic matrix can be impaired in different ways causing various diseases; for example, hypophosphatasia, where low ALP activity leads to low levels of inorganic phosphate. Hypocalcemia or hypophosphatemia, on the other hand, are a result of low blood levels of calcium or phosphate, respectively [169]. Impaired mineralization can ultimately lead to the development of rickets (in children) or osteomalacia (in adults), i.e., the softening of bone, potentially causing skeletal deformities and severely influencing the quality of life of affected patients [169,170]. Currently, treatments for these disorders include the supplementation of vitamin D and/or calcium (hypocalcemia), administration of the phosphate-regulating FGF23 antibody burosumab (hypophosphatemia), and enzyme replacement therapy (hypophosphatasia). Additional supplementation with MtVs to current treatments might be beneficial to achieve enhanced mineralization and thus to counteract the softening of the bones. 

### 5.3. Potential Risk of the Application of MtVs

The mineralizing potential of MtVs also has a major drawback, when it occurs at unwanted locations in the body. This unwanted mineralization is featured in pathological conditions such as vascular calcification, often seen in diabetic, hypertensive, and/or chronic kidney disease patients [21,164,171,172]. For example, the mechanism of initiation and progression of vascular calcification has been shown to be similar to physiological bone formation [164,173]. A high extracellular phosphate concentration upregulates the inorganic phosphate transporter 1 (Pit1), raising intracellular levels of inorganic phosphate. This results in the activation of RUNX2, enhancing the osteogenic transition of VSMCs [163,164,174]. These osteogenic VSMCs produce MtVs that cause microcalcifications inside the vessel wall that play a pathological role in the onset and progression of vascular disease [164]. The similarity of the mechanisms of soft tissue calcification and bone mineralization indicate the need for extra caution when considering MtVs for therapeutic purposes in mineralization-related bone diseases.

## 6. Prospect for Therapeutic Applications of MtVs

**Figure 3:**
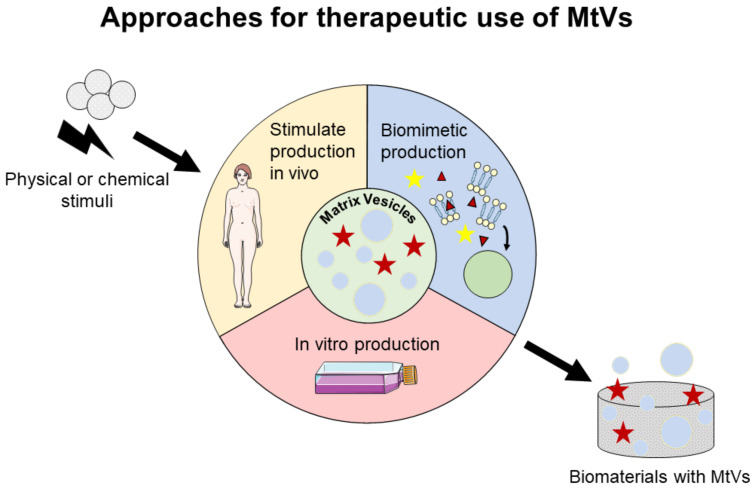
Possible approaches to use MtVs for therapeutics. MtV production can be stimulated in vivo by applying physical or chemical stimuli on MtV-secreting cells. Biomimetic or in vitro produced MtVs could be used in combination with biomaterials, for example. The figure was modified from Servier Medical Art, licensed under a Creative Common Attribution 3.0 Generic License (http://smart.servier.com/, accessed on 20 January 2021).

To use MtVs for therapeutic applications, we propose three approaches (Figure 3): (i) stimulate MtV secretion in vivo, particularly at the location where the mineralization is needed; (ii) stimulate MtV secretion in vitro by stimulating MtV-producing cells; and (iii) engineer biomimetic MtVs. For the second and third approaches, MtVs and biomimetic MtVs need to be delivered to the region of interest either by localized injection or biomaterial implantation. These approaches will be discussed further in the next sections.

### 6.1. Stimulation of MtV Secretion In Vitro and In Vivo

To maximize the potential of MtVs for desired mineralization, it is important to identify factors which can influence the characteristics and/or the composition of MtVs [175,176]. These factors could be used to guide MtV-producing cells to produce targeted MtVs in vitro and in vivo for therapeutics.

#### 6.1.1. Chemical Stimuli for MtV Secretion

Calcium and phosphate, as the main components of bone’s inorganic matrix, can trigger MtV secretion by MtV-producing cells. Treatment of osteoblasts with phosphate resulted in the secretion of MtV expressing high levels of ALP, Phospho1 and annexin A5, and containing hydroxyapatite crystals [134]. VSMCs and macrophages can also be stimulated with phosphate to induce MtV secretion which contained high levels of annexin A2, annexin A5, ALP and hydroxyapatite nucleation inside the vesicles [146,153,158]. Elevated calcium concentration, both extracellularly and intracellularly, can cause the secretion of MtVs and enhance mineralization as well [165,177]. Exposure of VSMCs to calcium ions led to the increased production of MtVs, which were accumulated by annexin A6 on the outer surface of MtVs, upregulation of PS, and formation of crystalline hydroxyapatite associated with both the outer and inner membrane of MtVs [178]. In addition, the concentration of intracellular calcium ions could be increased by ionomycin [127]. This facilitated the formation of calcium phosphate granules in the endoplasmic reticulum, and the transport to the mitochondria which resulted in the formation of MtVs intracellularly. 

The combination of calcium and phosphate has also been introduced to osteoblasts as a trigger for MtV secretion. For instance, synthesized calcium phosphate powders, comprising tricalcium phosphate and hydroxyapatite, induced the formation of spherical nodules containing calcium phosphate, indicating the formation of MtVs [179]. However, the formed MtVs were not fully characterized. Another study also demonstrated that the incubation of osteoblasts with hydroxyapatite nanoparticles resulted in the secretion of MtVs, as shown by TEM images [180].

The most biologically active vitamin D, 1α,25-dihydroxyvitamin D_3_ (1α,25-(OH)_2_D_3_), which acts through binding to the vitamin D receptor, promotes osteoblast differentiation of MSCs and primary osteoblasts in vitro and in vivo [181,182,183,184,185]. In vitro treatment of osteoblasts during the pre-mineralization phase with 1α,25-(OH)_2_D_3_ stimulates and accelerates matrix mineralization through increasing the number of secreted ALP-positive MtVs [186,187]. 

Other components capable of increasing MtV secretion are glycosaminoglycans (GAGs), which are key organic components of the extracellular matrix and play an essential role in the development of bone tissue. It has been shown that GAGs including hyaluronic acid (HA) and its synthetically sulfated derivatives could promote the osteogenic differentiation of MSCs and result in the secretion of MtVs [188]. Furthermore, treatment of osteoblasts with sulfated HA derivative had great influence on the proteome of MtVs [110]. The affected proteins such as thrombospondin-1 and -2, fibrillin-1, latent transforming growth factor β-binding protein 2, and fibronectin-1 can regulate vesicle–extracellular matrix interactions and MtV activity, leading to extracellular matrix formation and mineralization [110,189]. 

Besides the mentioned components, other factors have shown potential to enhance mineralization in vitro and in vivo [190]; thus, they can potentially stimulate MtV-producing cells to secrete MtVs. The secretion of MtVs under the influence of such chemical stimuli should be investigated further.

#### 6.1.2. Physical Stimuli for MtV Secretion

The secretion of MtVs under physical stimuli has not been thoroughly investigated, however stimuli such as mechanical forces, electrical and electromagnetic stimulation, ultrasound, shock wave and low-level laser therapy have been shown to promote bone regeneration in vivo and in vitro [191,192,193]. These physical stimuli led to increased osteogenesis and bone formation in vivo. For instance, pulsed electromagnetic field stimulation treatment reduced lumber vertebral osteoporosis by increasing bone formation and reducing bone resorption [194]. Whether these methods did induce the release of MtVs in vivo under physical stimulation has not been investigated. In vitro studies demonstrated that the application of these physical stimuli not only induced osteogenic differentiation, but also enhanced mineralization. This could be associated to the release of MtVs by the cells in culture. A recent in vitro study showed that static magnetic fields enhanced the osteogenic differentiation of MSCs and increased mineral formation through the release of MtVs into the extracellular matrix [195,196,197]. 

Overall, the MtV-producing cells can be chemically and physically stimulated, and induced MtV secretion in vitro. Secreted MtVs can be isolated and delivered to the site of interest via localized or systemic applications, for example, via injection. For both local and systemic delivery, a major challenge is to attain a suitable concentration of vesicles at the target site [144,198]. The use of biomaterials with integrated MtVs could help in delivering the vesicles to the targeted region in an appropriate concentration and allows for a controlled release of vesicles in this region. The in vivo application of chemical and physical stimuli has showed increased mineralization; thus, the release of MtVs can potentially be enhanced in the applied area. Further studies should investigate the in vivo secretion of MtVs under such stimuli.

### 6.2. Engineering Biomimetic MtVs

Apart from using cell-derived MtVs, one could also synthetically engineer biomimetic MtVs for therapeutic applications. These engineered MtVs give the possibility to fine-tune the cargoes and increase the specificity of MtVs to a certain region for targeted biomineralization. In the past few years, several attempts have also been made to create MtVs to better understand MtV-mediated mineralization. For example, the incorporation of ion carriers to transfer phosphate and calcium ions into triblock copolymer vesicles has shown that it is possible to form minerals inside such vesicles [199]. These primary vesicles are still rather simple models, but the concept of mimicking MtVs artificially seems a promising strategy.

#### 6.2.1. Proteoliposomes Mimicking MtVs

Proteoliposomes are lipid membranes with incorporated proteins that have been used as a tool for lipid–protein interactions studies, biotechnological applications, and the modeling of biological membranes [200]. They have been used to mimic MtVs to unravel the enzymatic activity of these vesicles and their contribution to the biomineralization process [201]. Various different combinations of enzymes and proteins associated to MtVs, such as ALP, NPP1 and annexin A5, were inserted in the lipid membranes [122,202,203,204,205]. It was found that the lipid composition could influence the formation of minerals and the activity of the enzymes or proteins [206,207]. For instance, the addition of cholesterol to the lipid membrane of the vesicles had an influence on the incorporated ALP [208]. A higher concentration of cholesterol in the membrane resulted in the formation of a more rigid monolayer which hampered ALP incorporation. Nevertheless, the enzymatic activity of incorporated ALP was enhanced in membranes with higher concentrations of cholesterol [208,209]. Moreover, a recent study has shown that the presence of cholesterol and sphingomyelin in the membrane of ALP-harboring proteoliposomes promoted amorphous calcium phosphate precipitation in vitro [210].

#### 6.2.2. Polymeric Vesicles Mimicking MtVs

Lately, other strategies have been developed to mimic MtVs. In a recent study, high concentrations of serine were used to stabilize amorphous calcium phosphate (S-ACP), which was then mixed with polyethylene glycol (PEG) to form PEG-S-ACP nanoparticles [11]. These nanoparticles were added to polysorbate to form micelles carrying these nanoparticles to establish a model of MtVs carrying amorphous calcium phosphate in vitro. It has been shown that changes in pH and surface tension of these models caused two forms of minerals, including crystalline mineral nodules and amorphous calcium phosphate particles. When introducing these models to collagen fibers, the crystalline needle-shaped apatites deposited on the surface of the collagen fibrils led to extrafibrillar mineralization, while amorphous calcium phosphate particles released from MtV-mimicking micelles showed infiltration of particles into the fibrils and were deposited intrafibrillarly [11]. In another study, ultrasmall black phosphorus nanosheets were encapsulated in poly (lactic-co-glycolic acid) nanoparticles to develop biomimetic MtVs [211]. The surfaces of the nanoparticles were modified with osteoblast-targeting aptamers, which are single-stranded oligonucleotides that use distinct structures to specifically bind to target cells [212]. The aptamers direct the MtVs to bind to osteoblasts, whereas the black phosphorus enhanced the local concentration of inorganic phosphate which facilitated the mineralization process. These biomimetic MtVs have shown great potential in facilitating in vivo biomineralization and promoting new bone formation [211]. 

More research on engineered biomimetic MtVs could address several issues regarding the mineralization of organic bone matrix. Besides targeting mineralization, these biomimetic MtVs could serve as efficient tools for various applications, including studying enzymatic defects associated with mineralization-related bone diseases and the screening of small molecules capable of modulating enzymatic activity of MtVs for potential therapeutic applications. The therapeutic potential application of MtVs as described in the previous sections are summarized in Table 3.

## 7. Conclusions

MtVs are a subset of EVs that are secreted by, e.g., osteoblasts, chondrocytes, and odontoblasts, to directly induce mineralization of the organic matrix. Multiple biogenesis pathways are described for MtVs, and consequently, they are very heterogenous. Regardless of their biogenesis and heterogenicity, MtVs are all located in the extracellular matrix and all contribute to biomineralization through multiple mechanisms. In addition to their role in biomineralization, MtVs also feature innate bone-targeting potential and osteo-inductive properties. These features of MtVs imply their therapeutic potential in bone regeneration or for treating bone pathologies such as osteoporosis. The therapeutical use of MtVs is still in its infancy; therefore, further characterization of MtVs, their cargoes, and their biomineralization potentials are important. Finally, exploiting in vivo and in vitro approaches to generate MtVs including to design biomimetic MtVs will accelerate the research on MtVs for therapeutic purposes.

## Figures and Tables

**Table 1:** Cargo and function of EVs derived from bone cells.

Cell Source of EVs	Cargo of EVs	Function of EVs
Osteoblast	RANKL [22,97]; miR-1192, miR-680 and miR-302a [93]; miR-125b and miR-503 [102,103].	Supports survival of osteoclasts in vitro and neo-osteoclastogenesis in vivo; Promotes osteogenic differentiation, as manifested by the up-regulated expression of osteogenic marker genes RUNX-2 and ALP, as well as enhanced matrix mineralization; Have anti-osteoclastogenic activity.
Osteoclast	RANK, EpCAM, CD63 [96,99]; miR-214 [95,101].	Maintain bone homeostasis through the RANK–RANKL interaction; Inhibits osteoblast activity in vitro.
Osteocyte	LAMP1, sclerostin, RANKL, and OPG [41]; miR-218 [106].	Attenuate bone formation in vivo; Inhibits sclerostin and influences the differentiation of osteoblasts.

RANKL, receptor activator of nuclear factor kappa-B ligand; miR, microRNA; RUNX-2, runt-related transcription factor 2; ALP, alkaline phosphatase; RANK, receptor activator of nuclear factor kappa-B; EpCAM, epithelial cell adhesion molecule; LAMP1, lysosomal-associated membrane protein 1; OPG, osteoprotegerin.

**Table 2:** Different types of MtVs and their physical, biological, and functional properties.

Biogenesis	Physical Properties	Biological Properties	Functional Properties
Ectosome-like	~50 nm–1 μm	- Rich in PS [114] - Rich in Annexins [115,116] - Exhibit membrane proteins and phosphatases from their parent cells (e.g., ALP, NNP-1) [18] - Accumulate calcium and phosphate internally upon release from their parent cell [18] - Calcium and phosphate can crystalize into HAp which can grow and disrupt the vesicle’s membrane to form a nodule [20]	Most likely secondary or extrafibrillar collagen mineralization
Exosome-like	~30 nm–150 nm	- Receive calcium and phosphate intracellularly from the ER via mitochondria [7,125,127] - Likely transported to the matrix by lysosomes that provide an acidic environment [126,127] - Acidity prevents crystallization of ACP [126]	Primary or intrafibrillar and secondary or extrafibrillar collagen mineralization [127]
Apoptotic bodies	~1 μm–5 μm	- Rich in PS [129] - Accumulate calcium and phosphate externally [130]	Vascular calcification [128] and Endochondral ossification [128,129,131]

MtV, matrix vesicle; ALP, alkaline phosphatase; NNP, nucleotide pyrophosphatase; PS, phosphatidylserine; ACP, amorphous calcium phosphate; HAp, hydroxyapatite.

**Table 3:** Approaches for therapeutic uses of MtVs.

Possible Approaches to Use MtVs	Methods of MtV Secretion or Production	Methods for Delivery of MtVs
Stimulate MtV secretion in vivo	- Physical stimulation [194]	- Locally applied on the region of interest
Stimulate MtV secretion in vitro	- Physical stimulation [197] - Chemical stimulation [134,186,188]	- Localized injection - Biomaterial implantation
Engineer biomimetic MtV	- Proteoliposomes [201] - Polymeric vesicles [11,211]	- Localized injection - Biomaterial implantation
